# Sensorless Estimation Based on Neural Networks Trained with the Dynamic Response Points

**DOI:** 10.3390/s21206719

**Published:** 2021-10-10

**Authors:** Omar Rodríguez-Abreo, Francisco Antonio Castillo Velásquez, Jonny Paul Zavala de Paz, José Luis Martínez Godoy, Crescencio Garcia Guendulain

**Affiliations:** 1 Industrial Technologies Division, Universidad Politecnica de Queretaro, El Marques 76240, Queretaro, Mexico; jose.martinez@upq.mx; 2 Red de Investigación OAC Optimización, Automatización y Control, El Marques 76240, Queretaro, Mexico; francisco.castillo@upq.mx (F.A.C.V.); jonny.zavala@upq.mx (J.P.Z.d.P.); crescencio.garcia@tec.mx (C.G.G.); 3 Information Technology Division, Universidad Politecnica de Queretaro, El Marques 76240, Queretaro, Mexico; 4 Tecnologico de Monterrey, School of Engineering and Sciences, Altamira 89600, Tamaulipas, Mexico

**Keywords:** Neural Network, parameter estimation, dynamic response, sensorless estimation

## Abstract

In the present work, a neuronal dynamic response prediction system is shown to estimate the response of multiple systems remotely without sensors. For this, a set of Neural Networks and the response to the step of a stable system is used. Six basic characteristics of the dynamic response were extracted and used to calculate a Transfer Function equivalent to the dynamic model. A database with 1,500,000 data points was created to train the network system with the basic characteristics of the dynamic response and the Transfer Function that causes it. The contribution of this work lies in the use of Neural Network systems to estimate the behavior of any stable system, which has multiple advantages compared to typical linear regression techniques since, although the training process is offline, the estimation can perform in real time. The results show an average 2% MSE error for the set of networks. In addition, the system was tested with physical systems to observe the performance with practical examples, achieving a precise estimation of the output with an error of less than 1% for simulated systems and high performance in real signals with the typical noise associated due to the acquisition system.

## 1. Introduction

The estimation of parameters is a widely studied problem; there are multiple works such as [1,2,3,4,5,6] in which the estimation of the parameters of the Photovoltaic Models is developed as the main object of study. It is due to the high interest in having a function that describes the dynamic behavior of the systems since its performance can be estimated as in [7], where the authors propose a sensor-less prediction system. Another option to use parameter estimation is precise control design since it is generally done theoretically based on system dynamics, as in [8,9,10,11,12] where the authors base the control design on the analysis of the dynamic model and the parameters precision gives the controller precision.

A recurrent option in the control and analysis of the dynamic system is the use of the Transfer Function. This type of function allows a general analysis of the system. The main advantage is that different systems can be represented in the same way, especially when studying first-order and second-order systems. Several investigations use systems of first-order or second-order [13,14,15]. The investigation [16] uses the parameter estimation of the Transfer Function instead of working with the dynamic model of the system to solve the inverse heat conduction problem. Another example of the use of Transfer Function is the work [17], where the authors estimate the dynamic behavior of the electrohydraulic servo drive through the Transfer Function.

There are various techniques for parameter estimation. One option is a heuristic method like the one presented in [18], where a parametric estimation system with the vector-type recursive least squares (VRLS) is developed. The investigations [19,20,21,22] use a heuristic method to estimate and control the systems based on the dynamic response. Another example for the analysis with a heuristic method is the study [23] where the authors do the Transfer Function parameter estimation with the Vector Fitting technique.

Another option for parametric estimation is the so-called population-based metaheuristic algorithms, such as the research presented in [24,25,26]. The authors use the Cuckoo Search Algorithm to estimate different types of physical systems. These types of algorithms have numerous advantages, among them their easy implementation [27], which allows them to be implemented in systems of multiple natures [28,29]. However, its nature is inherently iterative; therefore, its processing time is usually longer, and its implementation online is more complicated.

Some authors have developed adaptive techniques for parametric estimation. For instance, in the research shown in [30], the authors develop a method for parameters that vary in time. Another widely studied option is the use of Neural Networks. Several works exploit the use of networks in different fields [31,32]. Particularly in the field of parameter estimation, Neural Networks have shown excellent results, for example, in motors [33]. Another example is the work exhibited in [34], which showed the advances in parametric estimation through a Neural Network for lithium batteries.

Some authors have developed adaptive techniques for parametric estimation. For instance, in the study [30], the authors develop a method for parameters that vary in time. Another widely studied option is the use of Neural Networks. Several works exploit the use of networks in different fields [31,32]. Particularly in parameter estimation, Neural Networks have shown excellent results, for example, in motors [33]. Another example is the work exhibited in [34], which showed the advances in parametric estimation through a Neural Network for lithium batteries. Despite the different estimation and optimization methods, it is important to note that no one algorithm is better in all cases since, according to the No Free Lunch theorem, the algorithms cannot outperform the others if they are averaged over all possible problems. Much of the performance of the algorithms lies then in the correct parameters selection that governs their behavior and the problem specificity [35].

Unlike previous works, the proposed research is not based on estimating a specific system. Instead, it aims to develop a standard parametric estimation system for any open-loop stable systems using the second-order standard Transfer Function. Therefore, this method is applied to systems that enter a steady state with a finite value different than zero. The main contribution of this work is summarized in the estimation of the equivalent Transfer Function of second-order through Neural Networks for systems of any type of system based on the key characteristics of the dynamic response. This Transfer Function will allow estimating the response of the system and designing controllers effectively sensorless even if the system cannot be measured continuously or its parameters are difficult to calculate.

Additionally, this work presents the analysis of the components of the dynamic response as input of a Neural Network. The time rise and final value in steady state are the main factors of influence in estimating parameters based on the response to the step of a system. On the other hand, time delay and time peak are the variables that have a minor influence. Multiple architectures were analyzed to determine the best performance of the network, demonstrating that it can estimate the equivalent Transfer Function with only the key points of the dynamic response instead of all points. In addition to the vector of tests, the network was tested with two typical engineering systems: an electrical circuit and a DC motor to evaluate this network behavior with a step dynamic response in physical systems.

The rest of the work is described in the following way. Section 2 briefly describes the parts of the Transfer Function and their application in the description of physical systems. Section 3 shows the description of the Neural Network, its use, and the analysis of its performance based on the dynamic response. Section 4 presents the results of the trained network when implemented with signals from physical systems obtained through simulation. In Section 5 the analysis of the performance of the proposed network in real systems is analyzed. Finally, in Section 6, the conclusions are shown.

## 2. Second-Order Transfer Function Characteristics and Its Dynamic Response

The Transfer Function is highly used in control systems; it allows us to relate the input with the output of the system as expressed in Equation (1).

(1) $$G\left(s\right)=\frac{O(s)}{I(s)}$$

where G(s) is the Transfer Function, O(s) is the output of the system, and I(s) is the input. All variables are in the Laplace domain and are considered null initials values.

The function can be expressed in terms of the damping ratio (ξ), the natural frequency ($\omega_{n}$), and a DC gain (*k*), as seen in Equation (2).

(2) $$G\left(s\right)=\frac{O(s)}{I(s)}=k\frac{\omega_{n}^{2}}{s^{2}+2\xi \omega_{n}s+\omega_{n}^{2}}$$

The above equation represents a large number of physical linear and single-input single-output systems. Although these limitations in the representation of systems through a Transfer Function, motors, pistons, damping systems, among many other types of systems, can be represented with this expression. The behavior of the system output is calculated in a standard way if the system is stable in an open-loop for its analysis using a step type input of magnitude *a*. Therefore, the output of the system can be obtained by Equation (3).

(3) $$O\left(s\right)=k\frac{\omega_{n}^{2}}{s^{2}+2\xi \omega_{n}s+\omega_{n}^{2}}\frac{a}{s}$$

**Figure 1:**
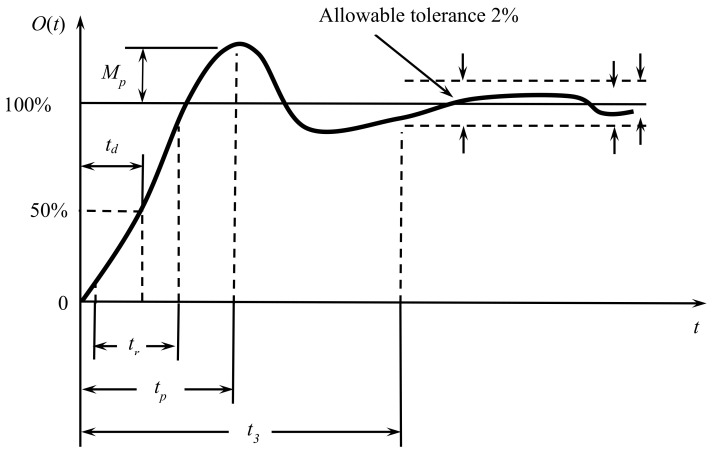
Key characteristics of the dynamic response to step input.

A step input generates a typical, expected dynamic response, as shown in Figure 1, where the key characteristics of the dynamic response are observed.

The function returns the characteristics in a structure containing the fields:

- Final value ($V_{f}$)—Value taken in the steady state;
- Rise Time ($t_{r}$)—Time it takes for the output to reach from 10% to 90% to the end value in steady-state response;
- Overshoot ($M_{p}$)—maximum peak value in percentage;
- Peak Time ($t_{p}$)—Time at which the overshoot happens;
- Time delay ($t_{d}$)—Time for reaching 50% of the final value the first time.
- Settling Time ($t_{s}$)—Time it takes for the error between the output and final value to fall within 2% of final value.

Each of these variables constitutes a part of the dynamic response for any system. However, depending on the type of response, some of these characteristics may not appear within the dynamic response. For example, overshoot only appears when the system is underdamped. In any other case, its value will always be zero. These characteristics are extracted and stored in a database as described in the next section. Finally, if the Final Theorem Value is considered, the value of *k* is determined by Equation (4).

(4) $$k=\frac{V_{f}}{a}$$

Equation (4) is valid only when time tends to infinity and the system is in the steady state. The value of *a* and $V_{f}$ are typically known. Therefore, the dynamic response of the system depends on ξ and $\omega_{n}$.

## 3. Neural Network as Parameter Estimator

Artificial Neural Networks (ANN) are widely used in any type of engineering problem, including control problems. The ANNs are mainly used for the resolution of non-linear systems. However, it can also be used in linear systems. However, they have not been tested as parametric estimators for Transfer Functions. This section explains the development of the Neural Network used for estimation tests.

**Figure 2:**
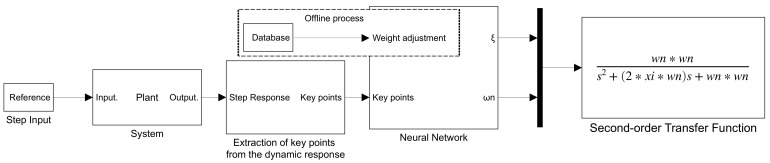
General process to implement the BP-ANN as parameter estimator of a Transfer Function.

The general process to use the network as an estimator is observed in Figure 2 and consists of training the Neural Network, subsequently using the step response to extract the key points of the dynamic response. With these points as input, the network estimates the parameters of the transfer function.

The first step is to select the type of architecture network, although, in the literature, there are multiple network architectures. The backpropagation (BP) Neural Network was chosen because it is the most widely used Neural Network [36]. The BP-ANN has been used in multiple types of systems, and their advantages and disadvantages are well known.

This type of network requires a training stage, and a broad database allows the network to know how the system responds to known inputs. For the trained and performance evaluation, an artificial database was created. It is observed in Equations (3) and (4) that two parameters must be adjusted to obtain different dynamic responses, the damping factor, and the natural frequency. The value of the amplitude and the gain *k* are directly related to the final output value of the system. Therefore, it is unnecessary to consider them as long as the value of the amplitude of the input step used is available.

**Figure 3:**
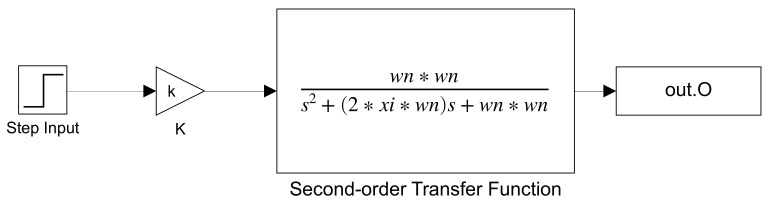
Simulation used for obtained the variables of the step response.

A total of 1,500,000 combinations were performed in which each parameter was varied within the range shown in Table 1 to cover a vast number of values and different dynamic response types. The Simulink model shown in Figure 3 was used to simulate the 1,500,000 combinations.

The parameter $\omega_{n}$ was varied uniformly to obtain 1000 values within the range. The case of ξ was treated with 1500 values since it has special cases. The underdamped case has ξ between 0 and 1, and 500 values uniformly distributed in that range were used. For the critically damped case, the value of ξ must be 1. The remaining values were uniformly distributed for the overdamped and high-overdamped cases.

The simulations were run, and the key points of the dynamic response and the values of ξ and $\omega_{n}$ were saved.

Once the database is completed, its data are filtered and all storage data without a finite response or with an establishing time longer than 100 s (22,234 data vectors) are removed. Subsequently, the architecture of the ANN was defined. The architecture depends mainly on four main parameters: the number of inputs and outputs, the number of hidden layers, the number of neurons, and the activation functions. Therefore, the next step is to select the inputs and outputs. The built database has several variables; the use in the ANN is described in Table 2.

Based on the tests and the equations of the dynamic response, it was decided to use independent networks for each of the outputs, that is, one BP-ANN for ξ estimation and one BP-ANN for $\omega_{n}$. Additionally, a pair of networks was used for critical damped, overdamped, and heavily overdamped systems and a different pair of networks for underdamped systems.

The division was carried out in this way because the overshoot is an important variable in underdamped systems. However, it will always be zero in other types of systems. Thus it cannot be considered a valid input for the critical damped, overdamped, and heavily overdamped network. Therefore, in this network, entry overshoot is removed from the network. There are several options for choosing the appropriate values for the remaining parameters of the architecture. However, it is necessary to carry out tests to analyze the performance of each proposed architecture.

Offline training was carried out using the filtered database. For this stage, the data were randomly divided to use 60% for training (886,670), 25% for validation (369,441), and 15% to carry out the performance tests (221,655). The training stage is crucial to determine the performance of the network. Training a Neural Network consists of adjusting each of the weights of the inputs of all neurons that are part of the Neural Network. Therefore, the responses of the output layer fit as closely as possible to the data we know. Typically, it is done by constructive methods, which allow the selection of the number of hidden neurons within the training process by evaluating the convenience of adding or not a new parameter to the network, depending on whether it increases the performance of the network.

The initial value of neurons was taken using the geometric pyramid rule. Later, the near values were evaluated according to the performance of the networks. The performance of the ANNs for this work was measured using the mean square error (MSE). The stop training conditions are 10,000 epochs, 30 validation checks, or gradient minor to $1\times 10^{-7}$.

Several architectures were tested during the hidden layers, the numbers of neurons per layer, and the activation functions. Transfer functions tested were Hyperbolic Tangent Sigmoid (Tansig), the Log-sigmoid (Logsig), and the Linear transfer function (Purelin). The most relevant tests for the architecture selection are shown in Table 3 for the system without overshoot. The overall performance of the pair of the networks is the average of MSE of both networks.

As seen in the previous Table, the N11a network has the architecture with the best performance in average. Therefore, the network used for systems without overshoot consists of two hidden layers with 10 and 16 neurons, respectively, with Tansgi-Tansig transfer functions for the hidden layers and Purelin for the output layer. Although architectures such as N12a offer similar performance with the same number of layers and neurons, the combination with these transfer functions offers the most considerable reduction in MSE, obtaining a 2.81% error in this architecture. On the other hand, the same tests were repeated for systems with overshoot. The results are summarized in Table 4.

The network N10b has the best performance considering the previous table. As in the previous case, the architecture with the lowest MSE was selected, in this case, $8.4\times 10^{-4}$%; for this they used two hidden capable with 8 and 12 neurons and Tansig transfer functions- Tansig between the hidden layers and Purelin for the output layer. A lower MSE can also be observed in general in networks used for systems with overshoot. Therefore, it was used for all subsequent tests when the system was underdamped. The previous results show an adequate performance of the network in estimating the Transfer Function parameters with reduced MSE values for $\omega_{n}$ and ξ parameters. However, it is desired to validate the inputs and their influence on the ANN to discard unnecessary or redundant entries since not necessarily all the listed characteristics impact the calculation of the parameters of the Transfer Function. Therefore, the network is retrained by deleting one input at a time to discover the effect of the inputs in the ANNs performance. The results of these tests in both sets of ANNs are shown in Table 5.

According to the results, the entry that contributes the most to MSE reduction is $t_{s}$ for overdamped systems and $M_{p}$ in underdamped systems. On the other hand, the most negligible impact on the network is $V_{f}$ with only 0.19% reduction to an error in overdamped systems and the $t_{d}$ in underdamped systems. However, its use is maintained since, in general terms, it benefits the ANNs and provides a positive effect even if it is small.

In concordance with the tests, the whole system used was the ANN N11a for critical damped, overdamped, and heavily overdamped systems with five inputs and two independent outputs and the ANN N10b for underdamped systems with six inputs and two separate outputs.

## 4. ANNs Evaluating Performance with Simulated Systems

The previous section only analyzes the error in the magnitude of the estimation of the parameters. However, it is necessary to examine how these errors in the Transfer Function parameters estimation affect the dynamic response from the point of view of the signals. Therefore, this section has explored the differences between signals produced by the set of ANNs and signals of simulated systems.

**Figure 4:**
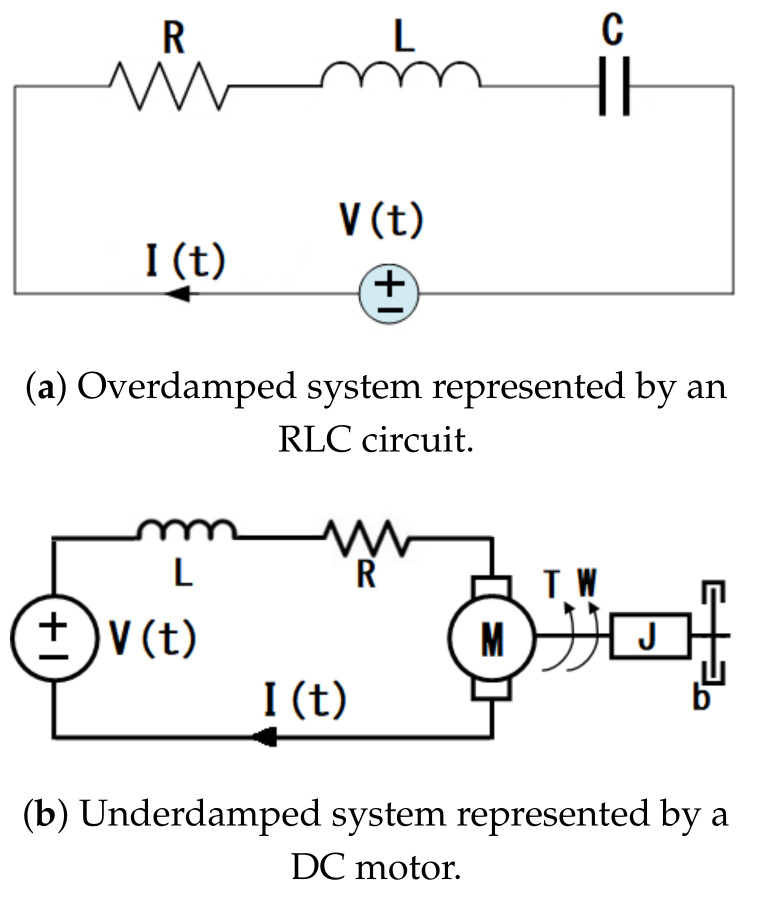
Systems used for the ANNs tests with real systems.

Two typical and well-known engineering systems were selected to test the ANNs signals. The Transfer Functions of an overdamped and underdamped system were taken into account. The overdamped system is the second-order electrical circuit shown in Figure 4a. The underdamped system is DC motor which is a widely used second-order electromechanical system, and its diagram is depicted in Figure 4b. The selected examples can be represented with their respective dynamic model. However, it is common to see them represented with their corresponding Transfer Function.

Figure 4a represents a second-order system and its Transfer Function, with the output as the voltage in the capacitor and the input as the voltage, is described by Equation (5).

(5) $$V_{c}\left(s\right)=\frac{1}{LCs^{2}+CRs+1}\frac{a}{s}$$

where $V_{c}$ is the voltage in the capacitor, L is the value of the inductor, and R is the value of the resistor. For the electromechanical system shown in Figure 4b, the output was considered as the angular velocity and the input is the voltage applied to the motor, and the Transfer Function is expressed in Equation (6).

(6) $$\omega \left(s\right)=\frac{K_{m}}{LJs^{2}+\left(RJ+LB\right)s+\left(RB+K_{m}K_{a}\right)}\frac{a}{s}$$

where V(t) is the voltage, *R* is the armature resistance, I(t) is the current in the mesh, $K_{e}$ and $K_{m}$ are the constant electrical and mechanical, respectively, *L* is the armature inductance, *J* is the momentum of inertia, and *B* is the friction coefficient. In practice, the constants $K_{e}$ and $K_{m}$ are similar and can be considered an equal constant of magnitude *K*. Therefore, Equation (6) can be rewritten as Equation (7):

(7) $$\omega \left(s\right)=\frac{K}{LJs^{2}+\left(RJ+LB\right)s+\left(RB+K^{2}\right)}\frac{a}{s}$$

**Figure 5:**
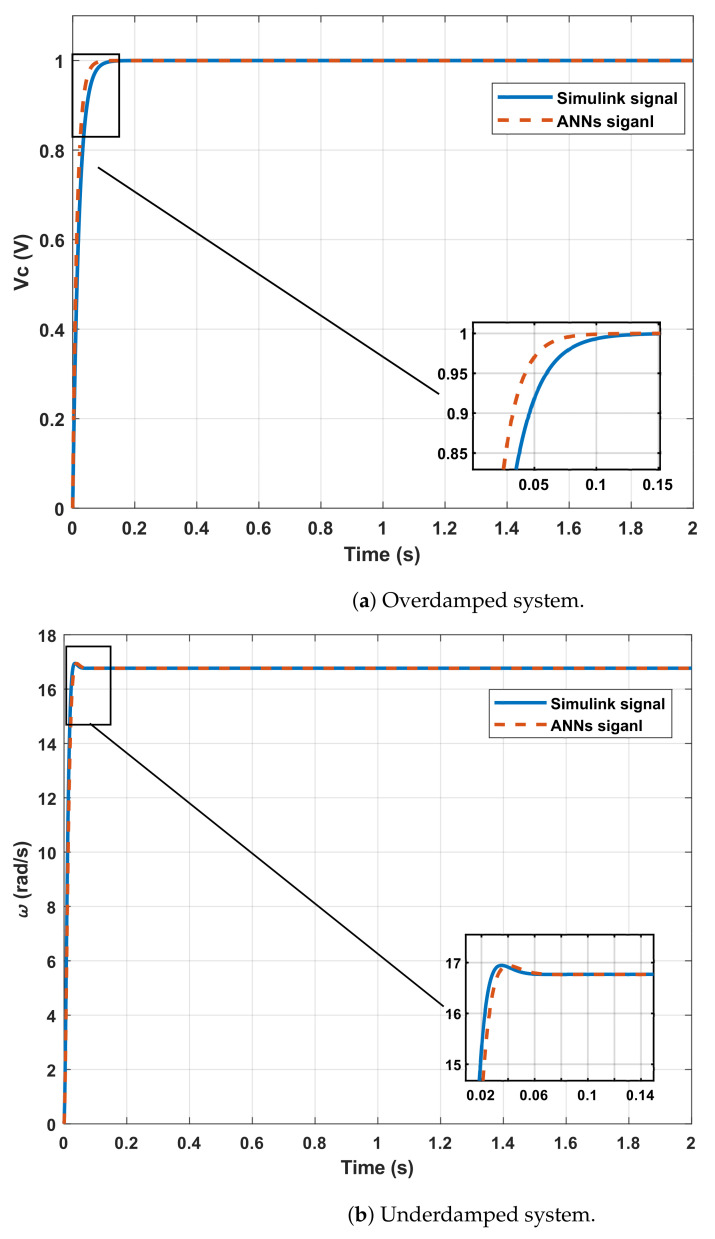
Systems used for testing the ANN with real systems.

The corresponding simulations were carried out to obtain the step response of each of the plants described above. The two systems were simulated by Simulink using the time and the values expressed in Table 6 and Equations (5) and (7). The outputs of both systems were saved, and the key points of the dynamic responses were extracted from these signals to use as ANNs sets inputs. The comparison between the signal calculated with the coefficients estimated by the ANNs and the simulated signals is shown in Figure 5.

The tests showed an MSE between signals of 0.45% for the overdamped system and 1.44% for the underdamped system. These results suggested that the parametric estimation can be given with a precision of less than 1.5% despite being of a different nature. The DC motor has a particularity of the $V_{f}$ that is greater than the magnitude of input *a*, but the use of the Final Value Theorem is helpful to process these types of systems.

Further, the results indicated that the errors in the estimation of the parameters in the Transfer Function are not significant in the response of the proposed system. It is also observed that the set of networks designed to estimate the parameters of systems with overshoots has a slightly better performance than the set of networks used for systems without overshoot. Although, this was an expected effect since in the network design phase this behavior was always shown.

## 5. Experimental Results

The system has been tested with simulated signals with satisfactory results. However, to verify the efficiency of the proposed method, the ANN performance is analyzed against a real signal. The DC motor shown in Figure 4b was taken as a reference to verify the performance of the proposed method with real systems. It was considered a Mavilor brand motor model CML 050 with the nominal parameters shown in Table 6.

The acquisition brings with it uncertainty problems due to the measurement hardware. In this case, the acquisition was made with the ADC (Analogue to Digital Converter) of the PIC18F4550 to measure the voltage input and a quadrature encoder to measure the angular velocity of the rotor.

**Figure 6:**
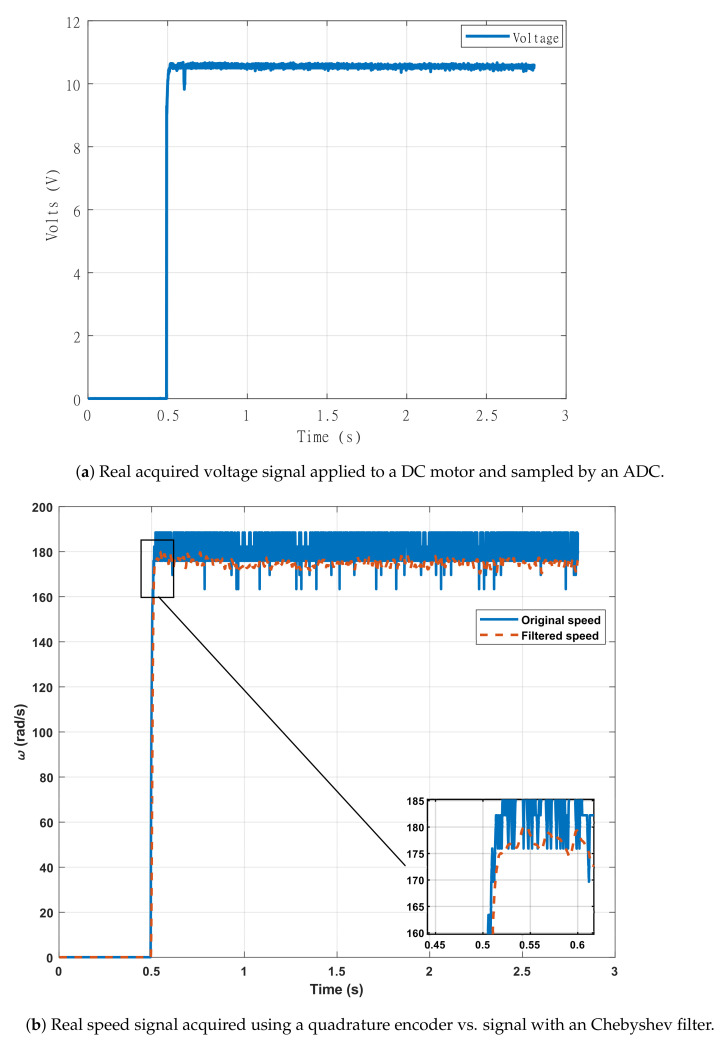
Systems used for test the ANN set with real systems.

The system is excited with a step input with a magnitude of 10.5 V to obtain the dynamic response and measured with the ADC, getting the signal shown in Figure 6a. For that, its speed data are sampled at a frequency rate of 1 kHz. Due to the ADC operating range, a signal conditioning stage is required since the motor operates with up to 24 V.

Each part of the conditioning and acquisition stage adds noise into the signal measured. The added noise is multifactorial and can be due to a low resolution in the sampling, the precision of the measuring instrument, or personal failures in using the instruments. Although noise can be reduced with more precise and accurate hardware, the real signals will always contain added noise that can also be reduced via software. Therefore, the work with real signals must consider filtering and averaging stages. In this sense, the original speed signal passed through a Chevicheb I filter. The result of the acquired signals is displayed in Figure 6b. On the other hand, to calculate the final or maximum values, the mean values were taken from three contiguous samples of the signal.

**Figure 7:**
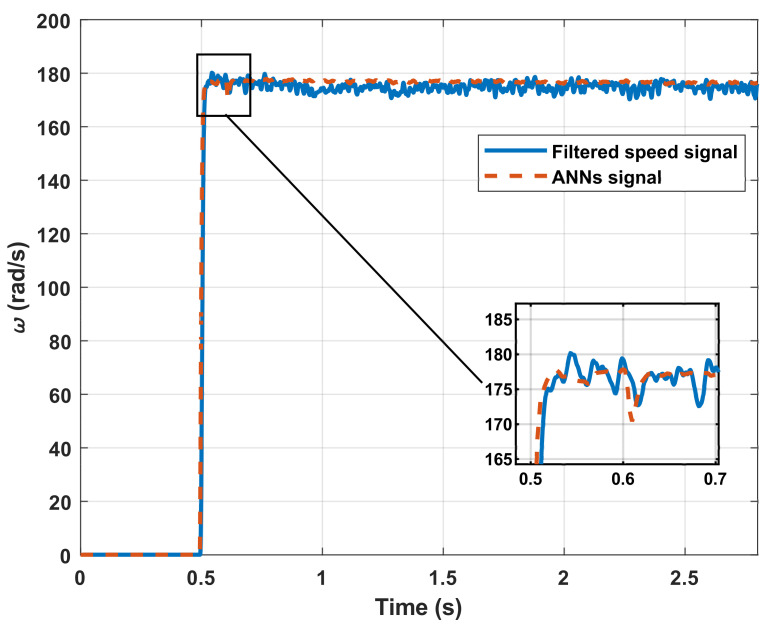
Simulation used for obtaining the variables of the step response.

The key characteristics of the dynamic response are extracted from the filtered speed signal (Table 7) and are used as inputs in the set of networks N11b. The result of the estimation is shown in Figure 7, where it is observed that the network provides an adequate response regardless of the step input magnitude with which the test is performed.

The error increases if it is compared with the data obtained in the simulation stages. This effect is also normal and was expected. However, the increase is due to the noise in the signals. Therefore, the system can be considered valid and capable of adapting to real work signals even with high noise. Nevertheless, it must be considered that the estimation precision depends not only on the network set precision but also on the accuracy of the measurement and adequate filtering of the signal. Table 8 compares the parameters obtained, the MSE between the original signal, and the signal estimated by the network developed to summarize the numerical results.

The MSEs for the DC motor shown in the previous table are normalized since their magnitude is different in each case. Hence, it goes through a normalization process to make an adequate comparison between the different MSEs.

## 6. Conclusions

In this work, a neural system was developed to estimate the parameters of a Transfer Function from the step response of the open-loop stable systems. Multiple tests were performed with different architectures to evaluate the best combination. Four BPNNs were used: two for the underdamped system and two for the overdamped systems.

The results indicate that the system can estimate coefficients of Transfer Functions in tests with simulated systems and real signals with an average error of 0.007% for the simulated signals and 0.1% for the real signal. According to Table 3, Table 4, and Table 8, there is a better performance for the set of networks in systems with overshoot because there is more information on the dynamic response than in systems without overshoot where it is just eliminated. Figure 5 and Figure 7, together with Table 8, show that the network reconstructions through the parameters estimated have an MSE of less than 1% for all cases.

The methodology presented in this work has the advantage of minimizing calculation time when the system is used online. Additionally, it is adaptable to any stable system, and the input is known (the same as in traditional parametric estimation). However, it has the disadvantages of the need for a database and training time for each network.

Unlike heuristic and metaheuristic methods, the parametric estimation was performed using only the characteristics of the respective dynamics of the system, avoiding comparing point to point to make a linear regression, allowing for the estimation of the performance of any system remotely even if its mathematical model is not known. BP-ANNs can correctly estimate underdamped and overdamped functions. This system can be adapted to practically any response signal with a larger database adapted to the expected ranges.

## Figures and Tables

**Table 1:** Ranges for variables used for generating the database.

$\omega_{n}$	ξ
$[1\times 10^{-6}$ 10,000]	$[1\times 10^{-6}$ 10,000]

**Table 2:** Variables of the database and its use in the ANN.

$t_{r}$	$M_{p}$	$t_{p}$	$t_{d}$	$t_{s}$	ξ	$\omega_{n}$
Input1	Input2	Input3	Input4	Input5	Output1	Output2

**Table 3:** Relevant tests for determining the architecture of ANNs for critical damped, overdamped, and heavily overdamped systems.

Network	Neurons	Activation	MSE	MSE	MSE
	in HL	Functions	$\omega_{n}$	ξ	Average
N1a	3	Tangsig-Purelin	4.529%	1.990%	3.260%
N2a	5	Tangsig-Purelin	4.138%	1.968%	3.280%
N3a	8	Tangsig-Purelin	4.421%	1.987%	3.204%
N4a	16	Tangsig-Purelin	4.506%	1.942%	3.224%
N6a	16	Tangsig-Tangsig	4.465%	1.990%	3.227%
N7a	16	Tangsig-Logsig	6.176%	15.871%	11.023%
N8a	5-3	Tangsig-Tangsig-Purelin	4.612%	1.987%	3.229%
N9a	12-8	Tangsig-Tangsig-Purelin	3.672%	1.987%	3.053%
N10a	8-12	Tangsig-Tangsig-Purelin	3.832%	1.994%	5.494%
N11a	10-16	Tangsig-Tangsig-Purelin	3.374%	1.989%	2.681%
N12a	10-16	Logsig-Logsig-Purelin	4.227%	1.994%	2.810%
N13a	10-16	Tangsig-Tangsig-Logsig	6.667%	15.79%	11.22%
N14a	14-20	Tangsig-Tangsig-Purelin	3.923%	1.980%	2.952%

**Table 4:** Relevant tests for determining the architecture of ANN in underdamped systems.

Network	Neurons	Activation	MSE	MSE	MSE
	in HL	Functions	$\omega_{n}$	ξ	Average
N1b	3	Tangsig-Purelin	1.001%	$4.1\times 10^{-3}$%	0.502%
N2b	5	Tangsig-Purelin	0.105%	$2.4\times 10^{-3}$%	0.054%
N3b	8	Tangsig-Purelin	$2\times 10^{-3}$%	$2.1\times 10^{-3}$%	0.002%
N4b	16	Tangsig-Purelin	0.070%	$1.8\times 10^{-3}$%	0.036%
N6b	16	Tangsig-Tangsig	0.304%	$1.9\times 10^{-3}$%	0.153%
N7b	16	Tangsig-Logsig	6.250%	4.180%	5.125%
N8b	5–3	Tangsig-Tangsig-Purelin	$9\times 10^{-3}$%	$1.9\times 10^{-3}$%	0.005%
N9b	12–8	Tangsig-Tangsig-Purelin	$3\times 10^{-3}$%	$1.9\times 10^{-3}$%	0.002%
N10b	8–12	Tangsig-Tangsig-Purelin	$1.7\times 10^{-3}$%	$3.25\times 10^{-4}$%	$8.4\times 10^{-4}$%
N11b	10–16	Tangsig-Tangsig-Purelin	$2\times 10^{-3}$%	$1.9\times 10^{-3}$%	0.002%
N12b	10–16	Logsig-Logsig-Purelin	$1.4\times 10^{-3}$%	$8.8\times 10^{-4}$%	0.001%
N13b	10–16	Tangsig-Tangsig-Logsig	4.209%	4.915%	4.562%
N14b	14–20	Tangsig-Tangsig-Purelin	4.182%	4.150%	4.166%

**Table 5:** Test for validation of the inputs of the ANN.

Input Removed	MSE Average	MSE Change	MSE Average	MSE Change
	for N11a	in N11a	for N10b	in N10b
$t_{s}$	3.071%	+0.39%	$1.6\times 10^{-3}$%	+$7.6\times 10^{-4}$%
$M_{p}$	-	-	$2.39\times 10^{-2}$%	+$2.3\times 10^{-2}$%
$t_{p}$	2.994%	+0.313%	$9.59\times 10^{-4}$%	+$1.19\times 10^{-4}$%
$t_{r}$	3.563%	+0.090%	$3.5\times 10^{-3}$%	+$2.7\times 10^{-3}$%
$t_{d}$	3.081%	+0.400%	$1.4\times 10^{-3}$%	+$5.83\times 10^{-4}$%
$V_{f}$	3.393%	+0.019%	$3.1\times 10^{-3}$%	+$2.2\times 10^{-3}$%

**Table 6:** Values used for simulate the plants.

Plant	Parameters Values	Time Simulation	Step Amplitude
RLC circuit	*R* = 20 Ω; *L* = 0.47 mH	2 s	1 V
	*C* = 100 μF		
DC motor	*R* = 3.13 Ω; *L* = 13.07 mH	2 s	1 V
	$B=169\frac{gm^{2}}{s^{2}}$; *J* = 9.0 μNm		
	K=0.0487		

**Table 7:** Variables of the dynamic response in real signal.

$t_{r}$	$M_{p}$	$t_{p}$	$t_{d}$	$t_{s}$	$V_{f}$
0.05 s	0.0241%	0.0430 s	0.010 s	0.004 s	178 rad/s

**Table 8:** Summary of the results.

	RLC Simulated	DC Motor Simulated	DC Motor Real
ξ estimated	46.141	0.8238 s	0.8225
$\omega_{n}$ estimated	$5.9\times 10^{3}$	137.06	160.1
General MSE in ANNs	2.681%	$8.4\times 10^{-4}$%	$8.4\times 10^{-4}$%
MSE between signals	0.014%	$1.44\times 10^{-4}$%	0.1%

## Data Availability

No new data were created or analyzed in this study. Data sharing is not applicable to this article.
